# Commentary on “p53 IHC Result as a Prognostic Tool in MDS”

**DOI:** 10.30699/ijp.2024.2027620.3295

**Published:** 2025-01-10

**Authors:** William Gesztes, Vahid Mehrtash, Victor E. Nava

**Affiliations:** 1 *Department of Pathology, The George Washington University Hospital, Washington D.C., USA*; 2 *Department of Pathology, New York University Grossman School of Medicine, New York, NY, USA*; 3 *Department of Pathology, Associate Professor, The VA Medical Center, Washington D.C., USA*

**Dear Editor,**

Recently, we had the pleasure of reviewing a paper entitled "p53 IHC Result as a Prognostic Tool in MDS," authored by Rezvani et al. and published in your July 2023 edition. The study investigated p53 protein expression in myelodysplastic syndrome (MDS) to better prognosticate patient outcomes. While the results provide strong evidence for p53 protein expression as an adjunct to prognosticate survival in MDS, there are certain considerations that merit closer inspection and discussion.

Important findings necessitate that fellow researchers replicate studies. However, the lack of certain methodological details in this study makes such efforts challenging. Firstly, the paper states that p53 analysis was “carried out according to the standard protocol.” A declaration such as this warrants, at a minimum, citation(s) referring to a ‘standard protocol.’ The authors discuss how prior studies have applied different percentage cutoffs when analyzing p53 expression in bone marrow. The application of a 1% threshold to differentiate between “positive” and “negative” p53 expressors in this context may appear arbitrary without a thorough explanation of how the authors agreed to this specific cutoff. If the threshold was data-driven by the study findings, this needs to be stated clearly. Critics may otherwise rightfully ask what the IHC analysis shows if a 2%, 5%, or higher cutoff is applied instead. Furthermore, p53 IHC staining inherently involves various degrees of nuclear intensity (i.e., 1+ light, 2+ medium, 3+ maximum), which may have implications independent of percentage expression, as has been shown in other organ systems, including the prostate (1–3). While nuclear intensity does tend to correlate with percentage expression, failure to address the intensity in this context invites valid criticism when calibrating a threshold. For example, it remains unclear whether light (1+) intensity nuclei are incorporated into the final, total percentage, as low-intensity nuclei are arguably not “overexpressed” per se (1, 2). Alternatively, is the cell count limited to only nuclei exhibiting maximum (3+) intensity? Clarifying this distinction is essential for accurate interpretation.

In the context of acute myeloid leukemia (AML), it has been demonstrated that p53 protein expression serves as an indicator for detecting TP53 mutations. p53 immunohistochemistry (IHC) has been validated as a rapid, cost-effective tool for identifying impactful TP53 mutations in AML, with a proposed 7.2% cutoff for p53-high as a key indicator, utilizing digital image analysis. Based on this observation, Tashakori et al. proposed a 10% or higher cutoff to consider p53 IHC staining as positive for reflecting TP53 gene mutations, which is significantly higher than the 1% cutoff used in this paper (4).

Additionally, it would have been valuable if the authors had included representative histomorphological images of p53-expressing bone marrow cells in their publication. As the age-old adage states, “a picture is worth a thousand words,” and including images may help clarify this methodological point for readers.

While it is true that wild-type p53 expression is short-lived, the sampled cross-sections of tissue reflect a moment in time when the tissue was excised. In other words, the selected area represents a “snapshot” of p53 protein levels that fluctuates over time and explains its inherently patchy expression, even in “normal,” unaltered p53-expressing cells. This observation brings us to another problematic argument presented by the authors during their discussion, best encapsulated by the statement: “The true frequency of TP53 mutations is underestimated, but IHC overexpression of p53 is always a marker for a molecular alteration with a poor prognosis.” While the argument can be made that the true frequency of TP53 mutations is underestimated, declaring that p53 overexpression is always a marker for an underlying molecular alteration is misleading. The paper referenced by the authors (5) to support this claim, in turn, cites a third source that states, “…the IHC detection of p53 protein suggests an underlying mutation in the gene.” These nuances require precise language for a balanced discussion of p53 prognostication in MDS.

Another important consideration is that the survival analyses have shown a notable association between p53 expression status and mortality. The data reveal a statistically significant association between p53 expression and the IPSS-R score, indicating that higher IPSS-R scores are often associated with positive p53 IHC results. To conclusively determine the independent prognostic significance of p53, it is imperative to evaluate the adjusted hazard ratio while controlling for the IPSS-R value.

In conclusion, while the paper addresses an important topic and contributes significantly to the field of MDS pathology, further clarification of the issues raised herein would be highly valuable for its practical implementation.
